# Foreign Body in Interior of Eye

**Published:** 1872-12

**Authors:** 


Foreign Body in Interior of Eye.—Prof. S. D. Gross (*Mich. Un. Med. Jour.*) recommends the following "treatment when a foreign substance, lodged in the interior of the eye, can not be extracted. Where useful vision yet remains, and the foreign body is creating no irritation, the case should be kept under close observation, and the patient cautioned to seek aid upon the appearance of symptoms of irritation in the sound or uninjured eye. Where useful vision is destroyed, and the foreign body yet remains, giving rise to destructive inflammation, the eyeball should be extirpated to remove the cause of a probable sympathetic ophthalmia in the sound eye. Although dissection has shown that foreign bodies in the eye, as shot, pieces of iron, and gun caps, may become encysted, yet such an event is extremely rare, and does not afford immunity against future attacks of inflammation. As long, in fact, as the extraneous substance remains, it is liable at any time, whether free or adherent, to provoke suffering and disease, and to induce sympathetic inflammation in the sound eye." *Medical Cosmos.*

